# Regulation of Membrane Turnover by Phosphatidic Acid: Cellular Functions and Disease Implications

**DOI:** 10.3389/fcell.2019.00083

**Published:** 2019-06-04

**Authors:** Rajan Thakur, Amruta Naik, Aniruddha Panda, Padinjat Raghu

**Affiliations:** National Centre for Biological Sciences-TIFR, Bengaluru, India

**Keywords:** lipid signaling, membrane transceptor, endomembrane compartments, model organism, cellular neurobiology, photoreceptores

## Abstract

Phosphatidic acid (PA) is a simple glycerophospholipid with a well-established role as an intermediate in phospholipid biosynthesis. In addition to its role in lipid biosynthesis, PA has been proposed to act as a signaling molecule that modulates several aspects of cell biology including membrane transport. PA can be generated in eukaryotic cells by several enzymes whose activity is regulated in the context of signal transduction and enzymes that can metabolize PA thus terminating its signaling activity have also been described. Further, several studies have identified PA binding proteins and changes in their activity are proposed to be mediators of the signaling activity of this lipid. Together these enzymes and proteins constitute a PA signaling toolkit that mediates the signaling functions of PA in cells. Recently, a number of novel genetic models for the analysis of PA function *in vivo* and analytical methods to quantify PA levels in cells have been developed and promise to enhance our understanding of PA functions. Studies of several elements of the PA signaling toolkit in a single cell type have been performed and are presented to provide a perspective on our understanding of the biochemical and functional organization of pools of PA in a eukaryotic cell. Finally, we also provide a perspective on the potential role of PA in human disease, synthesizing studies from model organisms, human disease genetics and analysis using recently developed PLD inhibitors.

## Introduction and Historical Perspective

**FIGURE 1:**
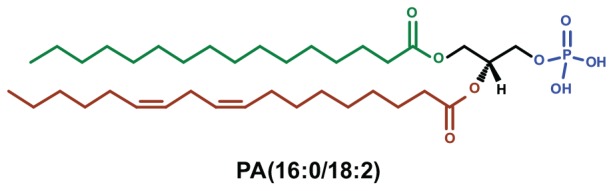
The chemical structure of phosphatidic acid. The glycerol backbone (black) of PA has esterified fatty acids at *sn-1* (green) and *sn-2* (red) position with carbon chain length of 16:0 and 18:2, respectively. The phosphate head group esterified at *sn-3* is shown in blue.

Phosphatidic acid (PA) is the simplest glycerophospholipid whose oldest known function is to serve as the backbone for the synthesis of a number of classes of glycerophospholipids. It consists of two fatty acyl chains esterified at positions *sn-1* and *sn-2* of glycerol and a free phosphate group at *sn-3* (Figure 1) reviewed in Athenstaedt and Daum (1999). Subsequently, it has become apparent that PA is also produced by biochemical reactions that are well understood as part of signal transduction pathways that mediate information transfer in eukaryotic cells. Through these pathways PA can mediate a diverse range of effects on eukaryotic cells that have been studied both in terms of basic cellular and molecular mechanisms and their potential involvement in disease processes. In this review we focus specifically on those functions of PA that relate to its ability to regulate membrane transport events in eukaryotic cells.

Compartmentalization into membrane bound organelles is a fundamental feature of eukaryotic cells (Rout and Field, 2017). Although the core principles of how membrane bound vesicles exchange material between the organelles of a cell have been known for some time (Pfeffer, 2013), there remains much interest in the mechanism by which this process is regulated. In this setting, the interest in the function of PA as a regulator of membrane transport rose from two strands of work. First, the study of secretion control in yeast had identified SEC14 as a PI/PC transfer protein required to support secretion and transport from the Golgi (Bankaitis et al., 1990). A genetic screen to identify suppressers and enhancers of *sec14* mutants had identified so called “bypass” mutants which encoded proteins involved in phosphatidylinositol (PI) and phosphatidylcholine (PC) biosynthesis (Cleves et al., 1991). Work in the Bankaitis lab uncovered the finding that for the bypass mutants to supress SEC14 function, yeast strains must have an intact SPO14 gene. SPO14 encodes phospholipase D (PLD), and enzyme that converts PC to PA (Sreenivas et al., 1998; Xie et al., 1998). Although SPO14 is a non-essential gene during vegetative growth, it is required for both prospore formation and PA production during starvation induced sporulation (Rudge et al., 1998, 2001); loss of spo14p leads to the accumulation of undocked membrane bound vesicles at the spindle pole body (Nakanishi et al., 2006). Subsequent elegant studies from the Neiman lab have shown that PA binds to spo20p, a v-SNARE required for fusion of vesicles to the prospore membrane (De Los Santos and Neiman, 2004; Liu et al., 2007). To date, these studies represent the most detailed analysis of a role for PA in regulating events in intracellular membrane transport in eukaryotic cells.

Secondly, in the context of metazoan biology, a role for PA in regulating intracellular membrane transport arose from two types of analyses (i) *in vitro* biochemical analysis which showed that small GTPases of the Arf family, known regulators of membrane transport can stimulate PLD activity (Brown et al., 1993; Cockcroft et al., 1994). (ii) Overexpression of PLD in multiple metazoan cells was able to modulate exocytosis (Vitale et al., 2001; Choi et al., 2002; Cockcroft et al., 2002; Huang et al., 2005), promote the generation of β-amyloid precursor protein containing vesicles at the TGN (Cai et al., 2006a). It was also shown that elevation of PA levels by multiple methods in *Drosophila* photoreceptors results in altered protein trafficking to the apical domain of these cells, collapse of the apical plasma membrane and the accumulation of endomembranes within the cell body (Raghu et al., 2009a). However, in contrast to the yeast system, until recently there had been limited evidence to support a role for PA in regulating intracellular transport in metazoan cells. A recent study has presented evidence supporting a role for endogenous PLD in regulating intracellular transport in *Drosophila* photoreceptors (Thakur et al., 2016).

## PA Synthesis and Turnover

**FIGURE 2:**
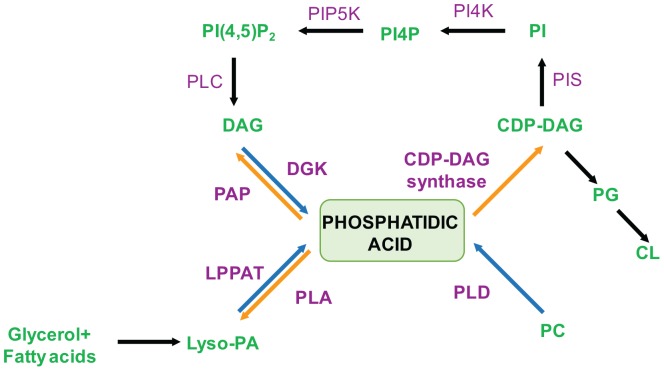
Schematic representation for biochemical pathways for the synthesis and metabolism of PA. Blue arrows indicate PA synthesis while orange arrows indicate turnover. Enzymes involved are marked in purple and the ones directly affecting PA levels are indicated in bold. Lipids species are marked in green. DAG, Diacylglycerol; CDP-DAG, Cytidine Diphosphate Diacylglycerol; PI, Phosphatidylinositol; PI4P, Phosphatidylinositol-4-phosphate; PI(4,5)P_2_, Phosphatidlyinositol-4,5-bis-phosphate; Lyso-PA, Lyso-phosphatidic acid; PC, Phosphatidylcholine; PG, Phosphatidylglycerol; CL, Cardiolipin; DGK, Diacylglycerol kinase; PAP, PA Phosphatase; LPAAT, Lyso-PA Acyl Transferase; PLA, Phospholipase A; PLD, Phospholipase D; PI4K, Phosphatidylinositol-4-kinase; PIP5K, Phosphatidylinositol-4-phosphate-5-kinase; PLC, Phospholipase C.

Cellular levels of PA are controlled in a spatiotemporal manner through the activity of multiple enzymes (Figure 2). These enzymes are located at distinct sub-cellular locations and use specific sources of substrate to maintain PA homeostasis and dynamics within cells.

The *de novo* synthesis of PA occurs by two acylation reactions wherein the first reaction leads to formation of monoacylated PA[also called lysophosphatidic acid (LPA)]. LPA formation can occur via one of two pathways; the first, seen in all organisms from bacteria to mammals utilizes glycerol-3-phosphate by the action of glycerol-3-P acyltransferase whereas the second occurs via the dihydroxyacetone phosphate pathway starting with the substrate dihydroxyacetone phosphate (DHAP). The LPA formed undergoes a second acylation catalyzed by lysophosphatidic acid acyl transferase (LPAAT). PA thus formed can be converted to diacylglycerol (DAG) by phosphatidic acid phosphatase (Carman and Han, 2009). DAG further serves as an intermediate in the biosynthesis of triacylglycerols and phospholipids like PC, phosphatidylethanolamine (PE) and phosphatidylserine (PS)that are important structural lipids. CDP-DAG synthase can also act on PA to form cytidine diphosphate diacylglycerol (CDP-DAG) that is also an intermediate in synthesis of various phospholipids like PI, phosphatidylglycerol (PG) and cardiolipin (CL) (Heacock and Agranoff, 1997).

The enzymes that generate pools of signaling PA are mainly PLD, diacylglycerol kinase (DGK) and LPAAT. PC-specific PLD hydrolyses PC to form membrane bound PA and free choline. PA thus formed performs various downstream signaling functions. Although PLD like genes are found in both prokaryotes and eukaryotes, in eukaryotes, in addition to the catalytic HKD motifs, a number of additional domains such as the PX, PH, myristoylation sequence and phosphatidylinositol 4,5-bisphosphate (PIP_2_) binding site are found that may serve to target the enzyme to specific membrane compartments reviewed in Selvy et al. (2011). While simpler eukaryote genomes contain a single gene encoding PLD activity, large and complex genomes such as those of mammals contain two genes PLD1 and PLD2 that biochemically show PLD activity [reviewed in Selvy et al. (2011)]. A recent study has suggested that the single PLD gene in *Drosophila melanogaster* encodes a protein that is functionally more similar to hPLD1 than hPLD2 (Panda et al., 2018). Though PLD1 and PLD2 are the most extensively studied, there are 4 other reported members of the mammalian PLD family, defined by the presence of a HKD motif. PLD3 and PLD4 are type II transmembrane proteins located at the ER and lysosomal compartments (Otani et al., 2011; Gonzalez et al., 2018). Although they belong to the PLD family, no canonical PLD enzyme activity has been reported. PLD5 is similar to PLD3 and PLD4 in that biochemical activity has not been demonstrated; a mouse knockout of PLD 5 has not shown any significant abnormalities (Karp et al., 2010). PLD6 or Mito PLD can hydrolyse cardiolipin on the outer membrane of mitochondria to generate PA (Choi et al., 2006). Along with this it has also functions as an endonuclease (phosphodiesterase) in piRNAs biogenesis (Watanabe et al., 2011).

It has been known since the 1980s that PLD is a signal activated enzyme in mammalian cells. Many agonists including hormones and neurotransmitters activate PLD [reviewed in Liscovitch (1991)]; interestingly many of these agonists also activate phospholipase C (PLC) resulting in PIP_2_ hydrolysis, a concomitant increase in intracellular calcium [Ca^2+^]_i_ and the production of DAG, an activator of protein kinase C (PKC). Interestingly, both Ca^2+^ and PKC have been studied as stimulators of PLD activity (Exton, 2002). In addition, small G-proteins of the Arf family appear to be required for full activation of PLD during GPCR signaling. A recent study has presented evidence that in *Drosophila* photoreceptors, where photons activate the GPCR rhodopsin leading to PLC activation, PLD dependent PA production also occurs but this does not requires Gq activity (Thakur et al., 2016). However, the biochemical steps leading to PLD activation during agonist mediated activation of G-protein coupled receptors (GPCR) remains unresolved.

Diacylglycerol kinases (DGK) are a family of lipid kinases that phosphorylate DAG to produce PA. DGKs are present in organisms from prokaryotes to mammals. In mammals, ten isoforms of DGK are reported that are grouped into 5 classes, each of which contains the DGK catalytic domain along with a range of additional domains that presumably lend both localization and regulatory properties [reviewed in Topham and Epand (2009)]. DGK activity is required to metabolize the DAG generated during receptor activated PLC signaling; loss of DGK results in enhanced PLC signaling based outputs in studies of multiple model systems (Rodriguez de Turco et al., 2001; Hardie et al., 2002; Zhong et al., 2003; Olenchock et al., 2006). Although direct evidence of a role for PA in phenotypes resulting from DGK deficiency have not been presented, it has been proposed that reduction of PA levels in *rdgA* mutants (diacylglycerol kinase in *Drosophila*) may result in transport defects to the apical membrane of photoreceptors (Suzuki et al., 1990). However, *laza* mutants (Type II PA phosphatase in *Drosophila*) that show elevated PA levels are able to suppress the retinal degeneration of *rdgA* mutants (Garcia-Murillas et al., 2006) suggesting that PA levels may be important for the phenotypes of DGK deficiency.

## Quantification of Phosphatidic Acid

Phosphatidic acid is a low abundance phospholipid in cells, being about two log orders less abundant compared to lipid classes such as PC; in biological samples PA is estimated to be 0.1–0.3 mole % of the total membrane lipids (Guan et al., 2013). Levels of PA can be estimated using multiple approaches such as thin layer chromatography (Holland et al., 2003), radionuclide (Munnik et al., 2000) or fluorescent labeling (Zhang et al., 2004). However, each of these methods has limitations ranging from low sensitivity to the inability to label total lipids in tissues. This poses a challenge in determining the levels of PA from tissue or cell samples without the benefit of radioactive labeling. Mass spectrometry has also been used to measure PA levels in tissues. The majority of published literature for PA analysis relies on triple quadrupole mass spectrometry coupled to reverse phase high performance liquid chromatography (HPLC) (Aaltonen et al., 2010; Bathena et al., 2011; Buré et al., 2013), although quantification of PA has also been reported using normal phase (Raghu et al., 2009a) and hydrophilic interaction liquid chromatography (HILIC) (Buré et al., 2013; Triebl et al., 2014). PA levels have previously been quantified from lipid extracts using the high resolution (100,000) and high mass accuracy (≤3 ppm) provided by Orbitrap technology (Yadav et al., 2015; Thakur et al., 2016). This method identifies lipids based on class specific chemical formulae generated from their monoisotopic mass; hence the exact composition of the fatty acyl chains cannot be determined. Recent work has established a new mass spectrometry-based method which can establish the fatty acyl chain composition of individual PA species and also quantify them. This method uses differential fragmentation patterns obtained from multiple reaction monitoring (MRM)-triggered information dependent (IDA) “on the fly” MS/MS’ (recorded as EPI) experiments, to establish *sn*-*1* and *sn*-*2* positional isomers present in PA from biological samples. This method should facilitate the analysis of PA in the context of cell signaling (Panda et al., 2018).

In addition to PA mass estimation, it is also informative to determine the spatial distribution of PA in cells. The spatial distribution of PA can be studied using PA binding domains (PABD) of proteins fused to fluorescent reporters. Such PABD are described below; some of the mostly commonly used PA probes are the PABD of SPO20 (Nakanishi, 2004), Opi1p (Loewen et al., 2004), PDE41A (Baillie et al., 2002), and Raf1 (Cells et al., 1996). In cells the PABD of SPO20 is localized to compartments like plasma membrane and nucleus (Nakanishi, 2004; Zeniou-Meyer et al., 2007) whereas the PABD of Opi1p is localized to the endoplasmic reticulum (ER) and nucleus (Loewen et al., 2004) and the PABD of PDE41A is found at the Golgi apparatus (Baillie et al., 2002). The differential localization of PABDs might be due to recognition of distinct pools of PA in a specific environment (Kassas et al., 2017) or additional regulators of their localization. However, these methods cannot give quantitative information about PA.

## Phosphatidic Acid Binding Module

Phosphatidic acid is a negatively charged lipid that regulates diverse cellular processes ranging from membrane trafficking to growth control (Jones et al., 1999; Foster, 2009). Some of these functions have been proposed to depend on its ability, as a cone shaped molecule, to alter lipid packing in a leaflet of the bilayer and thus membrane curvature. Many actions of PA are attributed to its ability to interact with PA binding proteins. Thus, in order to understand the *in vivo* regulatory functions of PA, it is important to study PA binding proteins. There have been various biochemical analyses primarily utilizing lipid affinity purification and LC–MS/MS mass spectrometry to identify novel PA binding proteins from tissue extracts (Manifava et al., 2001; Park et al., 2015). Such studies have revealed a broad range of PA binding proteins [reviewed in Raghu et al. (2009b); Stace and Ktistakis (2006)], however, in contrast to other lipid classes such as phosphoinositides that bind to specific domains (e.g., PX domain), to date no PA binding protein domain has been identified. Rather, it is thought that positively charged amino acids (e.g., lysine, arginine, and histidine) in PA-binding proteins interact with the negatively charged head group of PA (Stace and Ktistakis, 2006; Lemmon, 2008). PA-protein interactions can also be mediated by presence of the positively charged amino acids in well-defined domains of proteins like the PH domain of Sos (Zhao et al., 2007) or it can be in unstructured regions harboring several basic amino acids such as in the proteins Raf-1, mTOR,PIP5K, and DOCK2 (Fang et al., 2001; Stace and Ktistakis, 2006; Nishikimi et al., 2009; Roach et al., 2012). A recent review has highlighted factors that are likely to influence that ability of PA to bind to proteins given its unique physicochemical properties (Tanguy et al., 2018). Although a primary role for positively charged amino acids in mediating PA binding to proteins is central, the protonated state of PA, the presence of other zwitterionic lipids such as PE and the concentration of Ca^2+^ ions can also influence PA binding properties. The physicochemical properties of PA binding to proteins in the context of membranes is summarized in an excellent, recent review by Vitale et al. (2001); Tanguy et al. (2018).

## Phosphatidic Acid Functions

Phosphatidic acid is a cone shaped, low abundance membrane phospholipid (van Meer et al., 2008). By virtue of its shape, it can impart negative curvature to membranes and hence in principle influence membrane budding and fusion during vesicular trafficking. PA can also modulate membrane trafficking by binding to proteins that regulate various aspect of vesicular trafficking (Jones et al., 1999; Roth et al., 1999). Some of the important functions of PA in the context of membrane trafficking are described below:

### Receptor Transport

The ability of a cell to respond optimally to environmental changes is determined by the numbers and types of plasma membrane receptors. Upon ligand binding plasma membrane receptors like receptor tyrosine kinases (RTKs) and G protein coupled receptors (GPCRs) are activated and mediate the downstream signaling (Gether, 2000). Post-activation, these receptors are internalized either via clathrin mediated endocytosis (CME) (Wolfe and Trejo, 2007) or clathrin-independent endocytic mechanisms (Mayor and Pagano, 2007) or via fast-endophilin-mediated endocytosis (FEME) (Boucrot et al., 2015). Removal of cell surface receptors serves as a mechanism to regulate the levels of activated receptors on the surface and modulate the downstream signaling to a given ligand. The internalized receptors are subsequently degraded via lysosomes or recycled back to the plasma membrane (Irannejad and Von Zastrow, 2014).

Phosphatidic acid has been reported to play a regulatory role in CME (Antonescu et al., 2010). PLD activity itself has been implicated in trafficking and signaling from various membrane receptors (Exton, 2002; Selvy et al., 2011). Ligand induced endocytosis of EGFR requires PA generated by PLD1 (Lee C.S. et al., 2009). In presence of EGF, activated EGFR is internalized via CME with the help of the adaptor protein AP2 that recognizes EGFR via its μ2 subunit. In this context, it was seen that the PLD1 protein itself is an effector of PA and the auto-regulatory interaction between the PX domain of PLD1 and PA promotes the binding of PH domain of PLD1 with μ2 subunit and thereby facilitates EGFR endocytosis (Lee J.S. et al., 2009). PA also regulates the cell surface vs. intracellular distribution of inactive EGFR independent of the ligand. Inhibition of PA phosphatase activity causes acute increases in PA levels, inducing internalization of inactive EGFR in absence of ligand. It was seen that the internalization of inactive EGFR is through a PA effector-rolipram-sensitive type 4 phosphodiesterase (PDE4) that mediated down-regulation of PKA activity. The internalized EGFR accumulates in recycling endosomes and can either stay there without degradation for several hours or return to the cell surface when PA levels are reduced (Andres and Alfonso, 2010).

Micro-opioid receptors (MOPr) are a class of opioid receptors belonging to superfamily of seven transmembrane helix receptors. Activation of opioid receptors causes neuronal inhibition via multiple downstream effectors (Koch and Höllt, 2008). It has been shown that the agonist D-Ala2, Me Phe4, Glyol5-enkephalin (DAMGO) induced activation of MOPr also causes activation of PLD2 in an ARF dependent manner (Haberstock-Debic et al., 2003; Koch et al., 2003; Rankovic et al., 2009). MOPr and PLD2 physically interact with each other via the PX domain of PLD2 and regulate agonist-induced MOPr endocytosis (Koch et al., 2003). PLD2 activity has also been shown to be important for MOPr re-sensitization, as inhibition of PLD2 results in a decrease of agonist induced MOPr desensitization (Koch et al., 2004).

In neurons, class 1 metabotropic glutamate receptors (mGluR1 and mGluR5) are constitutively internalized via β-arrestin dependent and independent mechanisms (Sallese et al., 2000; Dale et al., 2001; Fourgeaud et al., 2003; Pula et al., 2004). PLD2 activity regulates the constitutive internalization of mGluR. It has been noted that PLD2 forms a complex with Ral and its guanine nucleotide exchange factor Ral-GDS. This novel complex constitutively interact with mGluRs by forming an adaptor and this agonist independent internalization does not appear to require β-arrestin (Bhattacharya et al., 2004).

In *Drosophila* photoreceptors, illumination activates the phototransduction cascade. Following light absorption, the GPCR Rhodopsin 1 (Rh1) undergoes photoisomerization to meta-rhodopsin (M). M is phosphorylated at its C-terminus, binds β-arrestin and this complex is removed from the microvillar plasma membrane via clathrin-dependent endocytosis to be either recycled back to the microvillar plasma membrane (Wang et al., 2014) or trafficked to the lysosome for degradation (Chinchore et al., 2009) [reviewed in Xiong and Bellen (2013)]. Tight regulation of this process is critical for rhabdomere integrity during illumination as mutants defective in any of the several steps of the rhodopsin cycle undergo light-dependent collapse of the rhabdomere [reviewed in Raghu et al. (2012) and see below]. During illumination, PA produced by dPLD regulates the recycling of Rh1 from late endosomal compartment in a ARF1 and retromer complex dependent manner back to the plasma membrane (Thakur et al., 2016). Hence during illumination, dPLD activity couples endocytosis of Rh1 loaded vesicles with their recycling to the plasma membrane thus maintaining plasma membrane composition and size. In summary, PA regulates the transport and signaling activity of several GPCRs by controlling their levels on the plasma membrane.

### Exocytosis

Phosphatidic acid produced by PLD activity plays an important role in regulating exocytosis. Early evidence implicating PLD in exocytosis emerged from studies of mast cells and neutrophils (Bader and Vitale, 2009). Ethanol, known to inhibit PA production by PLD, also inhibited exocytosis in mast cells stimulated via their high affinity Fc𝜀R1 receptor (Gruchalla et al., 1990) and degranulation in neutrophils (Korchak et al., 1988; Tou and Gill, 2005). Subsequently several studies have reported similar observations with regard to PLD activity and exocytosis in differentiated HL60 cells (Stutchfield and Cockcroft, 1993), sperm acrosome (Roldan and Dawes, 1993), adherent human polymorph nuclear leukocytes (Nakamura et al., 1994), pancreatic β-cells (Hughes et al., 2004) and neuroendocrine chromaffin cells (Bader and Vitale, 2009). PA generated via diacylglycerol kinase (DGK) has also been to shown to regulate release of azurophilic granules in anti-neutrophil cytoplasmic antibodies induced neutrophil exocytosis (Holden et al., 2011). Although these studies implicate PA in regulating exocytosis, mechanistic insights as to which specific step of the exocytic process might be regulated remains to be discovered.

### Phagocytosis

Phagocytosis is an essential process which enables immune cells like macrophages to internalize large particles (like extracellular particles, invasive pathogens, necrotic cells) into membrane-bound structure called the phagosomes (Niedergang and Chavrier, 2004). Such processes involve the ongoing extension of actin-rich protrusions and the consequent formation of phagosomes and macropinosomes (Flannagan et al., 2012). Lipids in general play a critical role in organizing various events of phagocytosis and PA also regulates multiple aspects of phagocytosis. In murine macrophages, PLD1 and PLD2 activity are necessary for efficient phagocytosis and PA is found to be transiently produced at the sites of phagosomes formation. In cells undergoing phagocytosis, PLD1 is recruited to nascent and internalized phagosomes, whereas PLD2 is only observed on nascent phagosomes. Thus both PLD isoforms are required for phagosome formation, but only PLD1 is implicated in later stages of phagocytosis occurring after phagosomal internalization (Corrotte et al., 2006). It was also seen that during phagocytosis, PLD2 forms a heterotrimeric protein complex with growth factor receptor-bound protein 2 (Grb2) and Wiskott-Aldrich syndrome protein (WASp). It is by virtue of this interaction that PLD2 can regulate the localization and activity of WASp. PLD2 anchors WASp to the cell membrane via Grb2 by protein-protein interactions and the PA produced by PLD2 leads to synthesis of PIP_2_ through PIP5K activity which in turn regulates the activity of WASp. This heterotrimeric interaction enables actin nucleation at the phagocytic cup and phagocytosis (Di Fulvio et al., 2007; Kantonen et al., 2011). In macrophages and dendrites, the basal PA required for constitutive membrane ruffling during micropinocytosis is primarily contributed by DGK and not by PLD activity (Bohdanowicz et al., 2013). PA is also known to regulate NADPH oxidase activity which plays important role in phagocytosis (Erickson et al., 1999; Palicz et al., 2001). Structural analysis of PX domain of the NADPH oxidase p47phox subunit by X-ray crystallography has identified two distinct pockets for phosphoinositide and PA binding (Karathanassis et al., 2002).

### Neuronal Function

Phosphatidic acid is proposed to play an important role in neurotransmission (Humeau et al., 2001; Bader and Vitale, 2009). PA is generated at the presynaptic ribbon terminals where it can regulate various steps of synaptic vesicle trafficking (Schwarz et al., 2011). PA produced by PLD has been shown to bind and modulate the activity of several proteins involved in synaptic vesicle endo and exocytosis such as NSF, PI4P5K, and syntaxin-1A (Manifava et al., 2001; Lam et al., 2007; Mima and Wickner, 2009; Roach et al., 2012). The interaction between PA and syntaxin 1A is believed to be necessary for regulating the energetics of membrane fusion (Lam et al., 2007). PA can bind and activate PIP5K (Moritz et al., 1992; Jenkins et al., 1994) to synthesize PIP_2_, an lipid important for neurotransmission and coupling of vesicular endocytosis to exocytosis at the synapse (Koch and Holt, 2012; Martin, 2015). Although there are number of studies linking PA produced by DGK to have a neuronal function *in vivo*, however, there is no direct evidence for the specific role of PA in the synaptic vesicle cycle (Tu-Sekine et al., 2015; Lee et al., 2016; Raben and Barber, 2017). In addition to multiple roles in the synaptic vesicle cycle, several studies have implicated PA produced by PLD1 and PLD2 in the intracellular trafficking of β-amyloid precursor protein (APP) and presenilin with important implications for amyloidogenesis (Cai et al., 2006a,b; Oliveira and Di Paolo, 2010; Oliveira et al., 2010b; Bravo et al., 2018). PLD1 is also reported to regulate autophagy mediated clearance of protein aggregates like p62 and Tau (Dall’Armi et al., 2010).

## Functional Organization of Signaling Pools of PA

Although numerous roles have been described for PA in regulating various aspects of cell biology, there are limited examples where the generation and functions of PA pools derived from multiple sources have been studied in a single cell type. One such cell type is the budding yeast *Saccharomyces cerevisiae* where metabolic labeling experiments and mutant analysis have tracked the generation and interconversion of PA pools [reviewed in Ganesan et al. (2016)]. These studies have primarily provided insights into the pools of PA involved in lipid biosynthesis.

**FIGURE 3:**
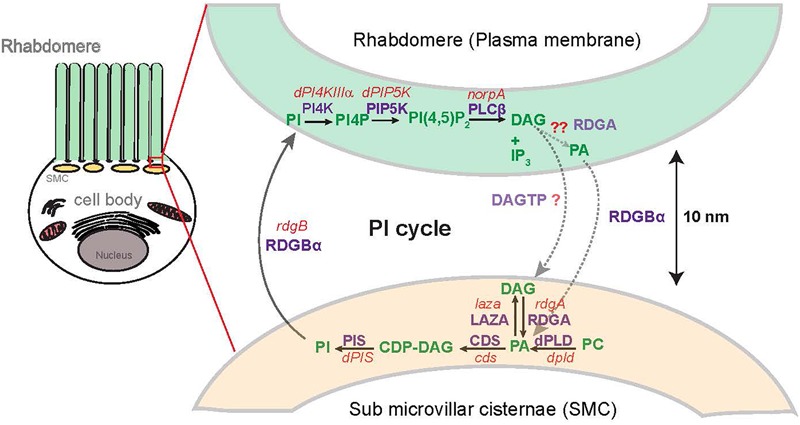
Spatial organization of the known pools of phosphatidic acid turnover in photoreceptors: Representation of *Drosophila* photoreceptor showing the spatial organization of lipid intermediate in the rhabdomere microvilli with respect to the sub-microvillar cisternae (SMC) and the cell body [Adapted from Yadav et al. (2018)]. Enzymes involved are mentioned in purple, gene names are mentioned in red and lipids involved are marked in green. The reactions marked in light green and light purple are proposed and experimental evidence remains to be established. PA, phosphatidic acid; DAG, diacylglycerol; CDP-DAG, cytidine diphosphate diacylglycerol; PI, phosphatidylinositol; PC, phosphatidylcholine; PIP, phosphatidylinositol 4 phosphate; PI(4,5)P_2_, phosphatidylinositol 4,5 bisphosphate; RDGA, diacylglycerol kinase encoded by the *rdgA* gene; LAZA-Type II PA phosphatase encoded by the *laza* gene; CDS-CDP-DAG, synthase encoded by the *cds* gene; dPLD, *Drosophila* PLD; PIS, phosphatidylinositol synthase.

By contrast, in *Drosophila* photoreceptors, the dynamics and functions of signaling PA generated by multiple enzymes has been studied in a single cell type allowing a synthesis of the control of cellular processes by multiple pools of PA (Figure 3). *Drosophila* photoreceptors are polarized cells whose cell biology is specialized for signal transduction during photon detection. During phototransduction, photon absorption by Rh1 triggers G-protein coupled phospholipase C (PLC) activity leading to a sequence of biochemical reactions during which PA is formed as a key intermediate. Mutants have been isolated and studied for several enzymes involved in PA metabolism in the context of signaling offering a setting in which the regulation and cellular functions of PA in a single cell type can be studied (Raghu et al., 2012).

Detailed genetic and biochemical analysis in *Drosophila* photoreceptors has shown that adult photoreceptors contain two main pools of PA generated by DGK (encoded by *rdgA*) and PLD (encoded by *dPLD*), respectively. Loss of either enzyme results in a reduction in levels of PA in adult photoreceptors (Inoue et al., 1989; Garcia-Murillas et al., 2006; Thakur et al., 2016). *Drosophila* photoreceptors also contain two enzymes that can metabolize PA; a single gene encodes CDP-DAG synthase activity (*cds*) (Wu et al., 1995); in *cds^1^*, a hypomorphic allele of *cds*, photoreceptors show elevated levels of PA during illumination confirming the requirement of this enzyme in metabolizing PA generated by DGK activity through conversion to CDP-DAG (Raghu et al., 2009a). In addition, photoreceptors also contain a Type II PA phosphatase activity (encoded by *laza*) that appears to be required for control of PA levels during illumination (Garcia-Murillas et al., 2006) and overexpression of *laza* further reduces the levels of PA in *rdgA^3^* photoreceptors (Garcia-Murillas et al., 2006). Together, these biochemical findings imply that DGK and Type II PA phosphatase can likely control the same biochemical pool of PA in photoreceptors.

*Drosophila* photoreceptors also contain a light stimulated PLD activity; loss of this PLD activity results in a reduction in PA levels (Thakur et al., 2016). Thus PLD activity contributes to PA levels in photoreceptors and since PLD knockout photoreceptors still express DGK, this observation implies that biochemically, these two enzymes contribute non-redundant pools of PA to photoreceptors. The PA levels in a *rdgA;dPLD* double mutant have not been described and it is presently unclear if at the biochemical level, there is any redundancy in the pools of PA generated by these two enzymes. These reduced PA levels can be rescued by either overexpression of RDGA or by loss of function of *laza* implying that PA arising from DGK activity can substitute for the loss of PA normally generated by PLD.

Since PA is a lipid, it is not freely diffusible across the cytoplasm and therefore restricted to the membrane at which it is produced. While the biosynthetic pool of PA is presumably generated at the ER membrane, signaling pools of PA are generated at membranes where the enzymes that generate them are localized; this would determine the spatial distribution of signaling PA. In *Drosophila* photoreceptors, phospholipase C is localized at the apical plasma membrane of photoreceptors and thus DAG is produced at this membrane. RDGA that phosphorylates DAG to generate PA is localized on the sub-microvillar cisternae (SMC). The SMC are a specialized ER derived membrane compartment that is located at the base of the microvillar membrane where it forms a membrane contact site (MCS) with the microvillar plasma membrane (Yadav et al., 2016). The importance of precisely localizing RDGA is underscored by the phenotype of *rdgA^1^*, the most severe allele of *rdgA*; *rdgA^1^* photoreceptors express normal levels of RDGA protein but an elegant immune electron microscopy study has demonstrated that the RDGA protein expressed in *rdgA^1^* photoreceptors is no longer localized to the SMC but distributed throughout the general ER in photoreceptors (Masai et al., 1997). Interestingly, PLD the other major source of signaling PA in photoreceptors is also localized to the region of the MCS between the plasma membrane and the SMC using immunofluorescence studies (Lalonde et al., 2005; Raghu et al., 2009a) although it is presently unclear at which of the two membranes the protein is localized; immunoelectron microscopy studies will be required to establish this point. The localization of endogenous LAZA in photoreceptors remains unknown; CDP-DAG synthase has been reported to be broadly distributed across the cellular ER in photoreceptors (Wu et al., 1995).

Functional analysis has also suggests that photoreceptors contain two major functional pools of PA. PA generated by RDGA, which is critical for normal electrical responses to light is generated in the context of G-protein coupled PIP_2_ turnover (Raghu et al., 2000; Hardie et al., 2002). Loss of RDGA function leads to deregulated lipid turnover during PLC mediated PIP_2_ turnover, excessive activation of TRP channels and retinal degeneration (Raghu et al., 2000; Hardie et al., 2004; Georgiev et al., 2005). From a cell biological perspective, retinal degeneration involves the collapse of the apical plasma membrane although the mechanism by which loss of RDGA and reduced PA levels leads to apical domain collapse remains unclear; Ca^2+^ influx through TRP channels is clearly an intermediate since retinal degeneration in *rdgA* mutants can be suppressed by loss of function mutants in *trp* (Raghu et al., 2000).

Loss of dPLD by contrast does not result in any detectable defects in phototransduction (Thakur et al., 2016) suggesting that this pool of PA does not contribute directly to PLC induced PIP_2_ turnover and TRP channel activation. Further, overexpression of dPLD in *rdgA* mutants does not suppress retinal degeneration suggesting that PA derived from PLD cannot support those sub-cellular processes normally underpinned by RDGA. The major function of PA derived from PLD activity is to support membrane transport processes associated with rhodopsin trafficking in photoreceptors. Recent work shows that in dPLD mutants Rh1 containing vesicles accumulate in the cell body following illumination. PA generated by dPLD seems to be required for the recycling of these rhodopsin containing vesicles back to the plasma membrane through the activity of the retromer complex [(Thakur et al., 2016) and see previous section]. Although the direct targets of PA that mediate control of vesicle recycling have yet to be identified, a role for Arf1, a known PA binding protein in this process has been proposed. In summary, the two major sources of PA in photoreceptors, DGK and PLD support distinct sub-cellular processes in photoreceptors.

Enzymes that metabolize PA have also been analyzed in the context of photoreceptor function. Hypomorphic alleles of *cds*, that encodes CDP-DAG synthase affect the electrical response to light (Wu et al., 1995) and also the re-synthesis of PIP_2_ during PLC signaling (Hardie et al., 2001). Independent studies using transmission electron microscopy have also demonstrated endomembrane defects in the photoreceptor cell body of *cds* mutants (Raghu et al., 2009a) and these defects appear to occur in the context of ongoing Arf1 activity under scoring the importance of CDP-DAG in controlling PA pools that regulate membrane transport. Thus CDP-DAG synthase is able to impact functions dependent on PA generated by both DGK and non-DGK sources.

LAZA, the Type II PA phosphatase is required to metabolize PA in photoreceptors generating DAG. *Laza* mutants show an altered electrical response to light (Kwon and Montell, 2006), are able to suppress the retinal degeneration of *rdgA* (Garcia-Murillas et al., 2006) and overexpression of *laza* enhances this phenotype (Garcia-Murillas et al., 2006). Therefore, LAZA is able to metabolize a pool of PA generated by DGK activity. *laza* mutants are also able to restore the levels of PA in *dPLD* loss-of-function mutants and also suppress the retinal degeneration seen in *dPLD* mutants (Thakur et al., 2016). Thus, a pool of PA controlled by LAZA is also able to regulate functions mediated by PA generated via dPLD activity.

**FIGURE 4:**
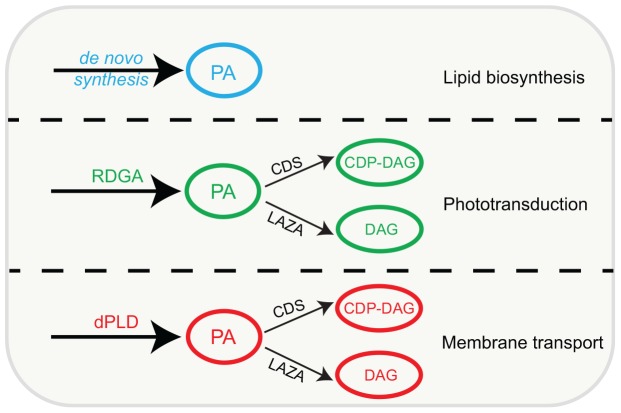
Model conceptualizing the major pools of PA in Drosophila photoreceptors. Individual, distinct pools are marked in specific colors, enzymes that can generate and metabolize these pools based on available experimental evidence are shown. PA, phosphatidic acid; DAG, diacylglycerol; CDP-DAG, cytidine diphosphate diacylglycerol; RDGA, diacylglycerol kinase encoded by the *rdgA* gene; LAZA, Type II PA, phosphatase encoded by the *laza* gene; CDS-CDP-DAG, synthase encoded by the *cds* gene; dPLD, *Drosophila* PLD.

In summary, while DGK and PLD generate biochemically and functionally distinct pools of PA, the enzymes that metabolize PA, namely CDP-DAG synthase and LAZA seem able to access both pools of this lipid in photoreceptors (Figure 4). The cell biological basis of how these pools of PA are segregated and support unique functions remains unknown and will be an interesting topic to analyze in the future.

## PA and Human Disease

### Infectious Diseases

Several studies have implicated cellular PLD activity in influencing the ability of viruses to enter and replicate in mammalian cells. Infection of respiratory epithelial cells with influenza virus is reported to stimulate PLD activity and chemical inhibitors of PLD2, RNAi depletion of PLD2 and pre-treatment with primary alcohols have all been reported to decrease the number of cells infected with viral particles and also the viral titer produced post-infection (O’Reilly et al., 2014; Oguin et al., 2014). Since PLD enzymes can form PtOH that is enriched in endosomal membranes and can influence membrane curvature, there has been interest in the idea that PLD activity can influence the ability of viral particles to enter cells and traffic through the endosomal system. PLD inhibitors have demonstrated anti-viral activity against HIV and also impact survival of intracellular parasites but the proposed mechanism of action does not appear to involve modulation of host trafficking systems.

### Central Nervous System

A number of studies in animal models have implicated PLD activity in the pathogenesis of stroke, injury, inflammation and neurodegenerative diseases of the central nervous system. Multiple mechanisms for these functions have been proposed [reviewed in Oliveira and Di Paolo (2010)]. In the context of the CNS, it is reported that PA produced by PLD activity can regulate the trafficking of amyloidogenic peptides (Cai et al., 2006a,b) and PLD2 ablation is reported to ameliorate synaptic dysfunction and cognitive defects in a mouse model of Alzheimer’s disease (Oliveira et al., 2010a). It has also been reported that rare variants in PLD3 confer risk for the development of Alzheimer’s disease (Cruchaga et al., 2014) and may do so via altering the levels of amyloidogenic peptides. However, a recent report using a mouse model of PLD3 has suggested that this may not be the mechanism of action although interestingly, this study also reported defects in the endo-lysosomal system in PLD3 mutants (Fazzari et al., 2017). Coffin-Lowry syndrome is a very rare form of X-linked mental retardation associated with growth and skeletal abnormalities^1^. A mutation in the protein Ribosomal S6 kinase 2 (RSK2) has been implicated as a cause of disease in some individuals with Coffin Lowry syndrome. Interestingly and pertinent to the topic of this review, phospholipase D has been reported to be phosphorylated by RSK2 and analysis in neural cell lines has suggested that this phosphorylation by RSK2 controls PLD1 activity and NGF induced neurite outgrowth; this study has proposed that PA may regulate vesicular transport in the growing neurite (Ammar et al., 2013, 2015). It has also been reported that the mRNA encoding diacylglycerol kinase kappa (DGKk) is one of the major RNA’s associated with the Fragile-X mental retardation protein (FMRP) in mouse cortical neurons (Tabet et al., 2016). Fragile X is the commonest form of inherited intellectual disability in children. Since the FMRP protein is thought to function by binding mRNA molecules and regulating their translation, FMRP is expected to control the levels of DGKk thereby tuning the switch of neurons between DAG and PA signaling states; molecular evidence for this was presented by Tabet et al. (2016) along with phenotypic similarities between the *Fmr1*^-/y^ mice and DGKk^-/-^ mice. It has been proposed that the switch between DAG and PA signaling may work via alteration in vesicular transport within dendritic spines (Moine and Vitale, 2019).

### Cancer

A number of studies have reported that PLD expression and activity are upregulated in a range of cancer types [(Bruntz et al., 2014; Kang et al., 2014) and references therein]. In genetically engineered mouse models, PLD1 can modulate tumor progression (Chen et al., 2012; Kang et al., 2015) and similar effects have been reported for PLD2 (Henkels et al., 2013; Wang et al., 2017). Since PA is the key product of PLD activity, it is possible that dysregulated PA signaling might contribute to one or more steps of cancer initiation or progression. A number of mechanisms are possible and some have been experimentally tested: (i) Since altered receptor tyrosine kinase signaling is a conserved feature of many cancers, it is possible that PA generated by PLD might contribute to tumor progression by propagating such signals (Henkels et al., 2013). In support of this idea one study has mapped the production of PA by PLD2 in relation to RTK signal transduction and shown its requirement for maintaining such signaling (Zhang et al., 2014). (ii) PA might contribute to the trafficking and secretion of factors that promote tumor progression; a potential role for PA generated by PLD2 in secretion of Type 1 Matrix metalloproteases, enzymes that are implicated in metastasis, has recently been presented (Wang et al., 2017). (iii) a third mechanism by which PA might play a role in cancer biology is through its ability to bind to and influence the mammalian target of rapamycin (mTOR) (Fang et al., 2001; Toschi et al., 2009), a key regulator of cell proliferation and growth. The source of PA that is sensed by mTOR has been debated; it has been suggested that PA generated by lipid synthesis rather than PLD/DGK signaling may be a nutritional signal in cells for mTOR (Foster, 2013) and experimental evidence to support this model has recently been presented (Menon et al., 2017). *De novo* synthesized PA is likely to contribute to membrane biogenesis and hence there are multiple mechanisms by which PA may contribute to cancer via altered membrane turnover.

### Human Genetic Disorders

With the development of modern methods of Next Generation Sequencing based genotyping, it has become possible to rapidly sequence and identify potential pathogenic DNA sequence variants in human genes of interest. In some cases, such variants show clear genetic transmissibility and the inheritance of such a variant can be clearly correlated with disease phenotype, strengthening the evidence implicating such variants in disease phenotypes. In the context of PA metabolizing enzymes, two such mutations have been reported. In the case of the PLD1 gene, studies have implicated mutations in the PLD1 gene in two families with congenital cardiac valvular defects (Ta-Shma et al., 2017). These mutations segregate with disease phenotypes and were assessed to have a functional impact through studies in model organism systems. In addition, a pathogenic variant in PLD3 that reduces PLD3 activity has been reported in a family with spinocerebellar ataxia (van Dijk et al., 1995; Nibbeling et al., 2017). Finally, mutations in DGKe have been reported to result in hemolytic uremic syndrome (Nephrotic syndrome Type 7) (Lemaire et al., 2013; Ozaltin et al., 2013). The cell biological and molecular mechanism by which these mutations in PLD and DGK lead to the phenotypes described in these human patients remains to be elucidated.

In addition to the aforementioned studies on individual human families with defined clinical features, variants in PLD1, PLD2 and most DGK isoform genes have been linked in Genome Wide Association Studies (GWAS) with a range of human phenotypes including several diseases of the brain, autoimmune diseases, physical traits such as body mass Index and metabolic disorders. A catalog of these variations and the studies in which they were analyzed can be found at https://www.ebi.ac.uk/gwas/. Detailed analysis using experimental models will be required to understand the specific roles of PA metabolizing enzymes in these contexts.

## Summary and Future Directions

Although numerous studies have implicated PA in the regulation of membrane transport and sub-cellular organization of compartments in eukaryotes, until recently, there has been limited progress in establishing the function of PA using model systems *in vivo*. With the recent availability of new genetic knockout models in multiple organisms including worms, flies, zebrafish and mice, is should now be possible to perform insightful studies into the function of PA in endogenous cell types. Further, novel ways of measuring PA in cells using fluorescent probes and mass spectrometry with the need for radiolabeling should facilitate such analysis *in vivo* using these genetic models. A number of key questions remain with respect to the signaling functions of PA:(i) As a signaling lipid how is the production and metabolism of PA coupled to ongoing cellular changes by controlling the activity of enzymes that tune its levels in cells? (ii) How does PA exert its effects on cells. Although a number of PA binding proteins have been described, the mechanisms by which it controls membrane transport remain poorly understood. PA can also be metabolized to lyso-PA and DAG both of whom may exert cellular effects by themselves and it has also been proposed that due to its cone shape, PA may also work independent of protein targets by altering local membrane curvature. Biophysical and biochemical studies using *in vitro* reconstitution will likely play a key role in answering these questions. These open questions represent interesting areas of analysis for the future. Finally, with the availability of high-throughput methods of NGS sequencing it should be possible to catalog variations in human PLD genes and link them to interesting phenotypic outcomes in health and disease.

## Author Contributions

All authors listed have made a substantial, direct and intellectual contribution to the work, and approved it for publication.

## Conflict of Interest Statement

The authors declare that the research was conducted in the absence of any commercial or financial relationships that could be construed as a potential conflict of interest.

## Footnotes

1 https://www.omim.org/entry/303600
